# *Trans–Cis* Kinetic Study of Azobenzene-4,4′-dicarboxylic Acid and Aluminium and Zirconium Based Azobenzene-4,4′-dicarboxylate MOFs

**DOI:** 10.3390/molecules27041370

**Published:** 2022-02-17

**Authors:** Refilwe Mogale, Jeanet Conradie, Ernst H. G. Langner

**Affiliations:** 1 Chemistry Department, University of the Free State, Bloemfontein 9300, South Africa; 2011118029@ufs4life.ac.za (R.M.); conradj@ufs.ac.za (J.C.); 2 Department of Chemistry, UiT—The Arctic University of Norway, N-9037 Tromsø, Norway

**Keywords:** *trans*–*cis* kinetics, metal organic frameworks, photo-responsive, dye removal

## Abstract

Metal organic frameworks (MOFs) are porous hybrid crystalline materials that consist of organic linkers coordinated to metal centres. The *trans*–*cis* isomerisation kinetics of the azobenzene-4,4′-dicarboxylic acid (AZB(COOH)_2_) precursor, as well as the Al^3+^ (Al-AZB)- and Zr^4+^ (Zr-AZB)-based MOFs with azobenzene-4,4′-dicarboxylate linkers, are presented. The photo-isomerization in the MOFs originates from singly bound azobenzene moieties on the surface of the MOF. The type of solvent used had a slight effect on the rate of isomerization and half-life, while the band gap energies were not significantly affected by the solvents. Photo-responsive MOFs can be classified as smart materials with possible applications in sensing, drug delivery, magnetism, and molecular recognition. In this study, the MOFs were applied in the dye adsorption of congo red (CR) in contaminated water. For both MOFs, the UV-irradiated *cis* isomer exhibited a slightly higher CR uptake than the ambient-light exposed *trans* isomer. Al-AZB displayed a dye adsorption capacity of over 95% for both the UV-irradiated and ambient light samples. The ambient light exposed Zr-AZB, and the UV irradiated Zr-AZB had 39.1% and 44.6% dye removal, respectively.

## 1. Introduction

Metal organic frameworks (MOFs) are 3D crystalline porous materials obtained by the self-assembly of metal ions with appropriate bridging organic ligands. The organic ligand azobenzene is well-known for its reversible *trans*–*cis* photo-isomerisation about the azo bond. The atoms attached to the azobenzene derivatives have an effect on the kinetic constant of isomerisation. Electron-withdrawing groups typically have a retarding effect on the rate of azobenzene isomerisation. A study on the influence of halogens on free azobenzene ligands showed that the rate of isomerisation decreases as electronegativity and electron-withdrawing strength increase: F (4) < Cl (3) < Br (2.8) [1]. A similar trend was observed by Bujak et al., in which electron-withdrawing groups retard the rate of isomerisation [2]. Although the electron-withdrawing groups retard the rate of isomerisation, they also extend the half-lives of azobenzene derivatives [2].

**Scheme 1:**
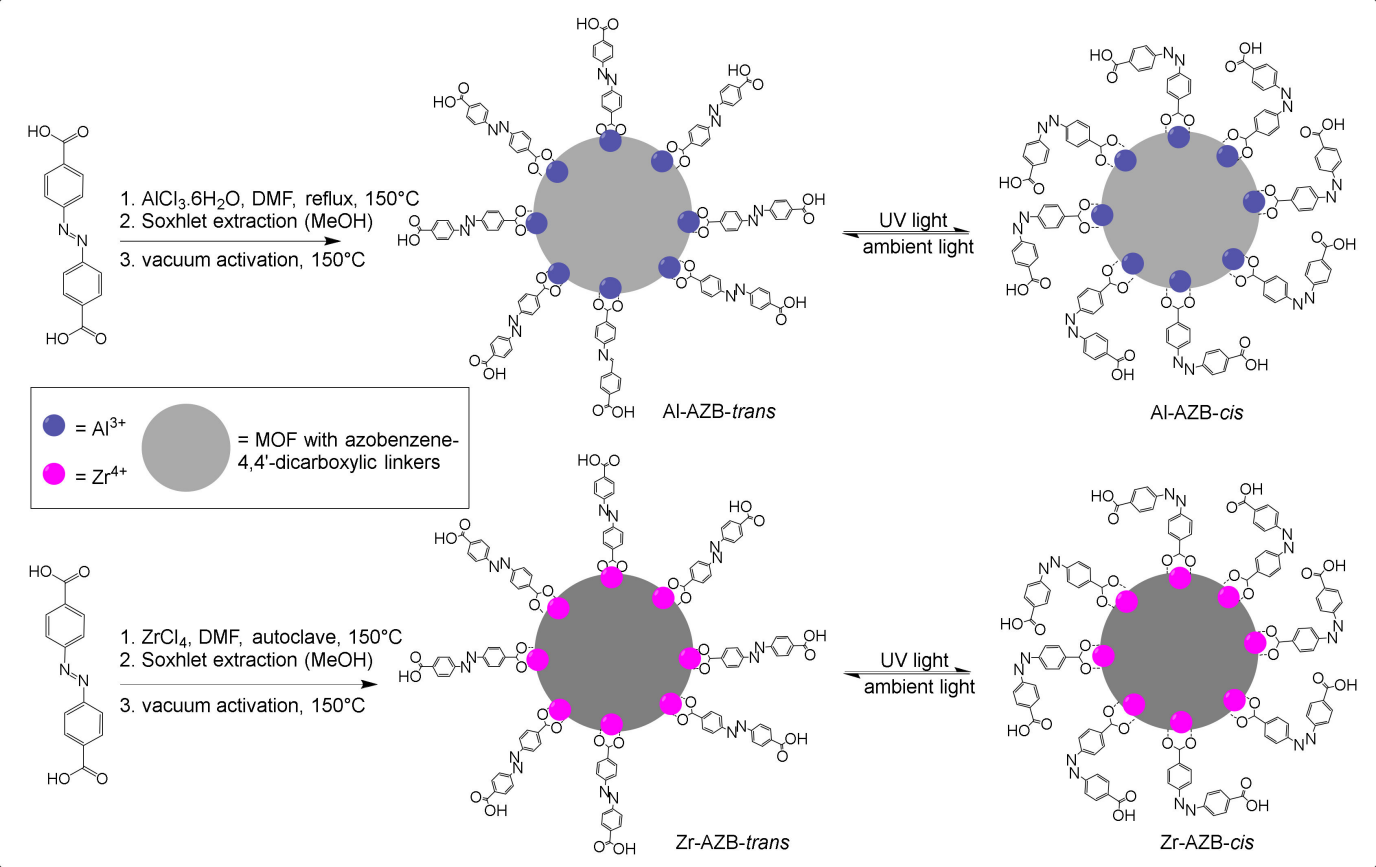
Synthesis of azobenzene dicarboxylate MOFs of this study [7], with a representation of the MOFs. Each Al (blue) is 6- and each Zr (pink) is 8-coordinated, extending into 3 dimensions (grey circles). Complete *cis* isomerization could not be obtained.

Azobenzene-4,4′-dicarboxylic acid is used as an MOF linker, e.g., in azobenzene-containing zirconium MOFs demonstrated to be effective heterogeneous catalysts for the direct amidation of benzoic acids [3]. Terbium and dysprosium MOFs with azobenzene-4,4′-dicarboxylic acid as linkers display intense photoluminescence properties in the solid-state [4]. The dynamic *trans*–*cis* photo-switching in zinc-based MOFs containing azobenzene-4,4′-dicarboxylate linkers lead to dynamic light-induced structural flexibility, which facilitates carbon dioxide capture and release [5]. The *trans*–*cis* azobenzene-photo-isomerisation ability also changes the pore environment in a Cu MOF (containing both homochiral D-camphoric acid and light-responsive azobenzene moieties), leading to the enantioselective adsorption of chiral molecules [6]. In all these applications, the rate of the *trans*–*cis* isomerization is of importance; thus, this study reports the *trans*–*cis* isomerisation kinetics of azobenzene-4,4′-dicarboxylic acid and azobenzene dicarboxylate MOFs with Al^3+^ and Zr^4+^ metal centres. The synthesis scheme as described in our previous work is shown in Scheme 1 [7]. Graphene, functionalised with azobenzene side groups on the surface shows similar photo-responsive behaviour as reported in this study [8,9]. Surface-mounted Cu MOFs (SURMOFs) with pillar linkers containing azobenzene side groups between the Cu atoms also show photo-switching, without reporting the kinetics thereof [6,10,11]. A theoretical study on Zr-AZB MOF-containing azobenzene linkers and singly bound azobenzenes on the surface focused only on the adsorption of gases [12]. The MOFs of this study contain azobenzene linkers and singly bound azobenzenes on the surface [7].

## 2. Materials and Methods

Azobenzene-4,4′-dicarboxylic acid, AZB(COOH)_2_, and the azobenzene-4,4′-dicarboxylic linker MOFs, Al-AZB and Zr-AZB, were synthesised and characterised as described in our previous work [7]. All chemicals were purchased from Sigma-Aldrich and Merck (Modderfontein, Johannesburg, South Africa) and the characterisation techniques are presented in the supplementary information (Supplementary Materials Figures S1–S3). For photophysical measurements (UV–Vis and fluorescence), the indicated concentration of the samples (MOFs and ligand) were suspended (ligand dissolved) in the indicated solvent (water/DMF/CHCl_3_) and then irradiated over time with a Spectroline UV lamp at a 365 nm UV wavelength (4 W, 230 V, 50 Hz, 0.17 A). UV–Vis measurements of the MOF suspensions were analysed on the Olis Clarity CCD (Georgia Atlanta, United States) UV–Vis Spectroscopic system (effective cell path length, 30 cm). Fluorescence measurements were performed using an Edinburgh Instruments FLS980 PL (Livingston, United Kingdom) spectrometer with double monochromators fitted with a 150 W CW ozone-free xenon lamp as an excitation source.

The *trans–cis* isomerisation reaction kinetics were monitored by UV–Vis spectrophotometry in water, DMF, and chloroform at 25 °C. The change in absorbance at the wavelength of the maximum absorbance was monitored for the *trans* isomer (294 nm ligand in water, 307 nm ligand in CHCl_3_ and DMF, 380 nm MOFs) and for the *cis* isomer (445 nm ligand in water, 413 nm ligand in CHCl_3_ and DMF, 457 nm MOFs). The observed first-order rate constants, *k*_obs_, were obtained by plotting ln(A_inf_ − A_t_) vs. time data according to the first-order kinetic equation:

ln(A_inf_ − A_t_) = −*k*_obs_t + ln(A_inf_ − A_0_) (1)

where A_t_ = absorbance of a complex at time t, A_inf_ and A_0_ are absorbances of a complex at times infinity and zero, respectively. 

The half-life of the *cis–trans* isomerisation was determined by Equation (2):

t_1/2_ = ln(2)/k (2)

The Tauc plot was plotted using Equation (3) [13,14]:

αE_p_ = K(E_p_ − E_g_’)^1/2^ (3)

*α* = 2.303 A/d (4)

E_p_ = hc/λ (5)

where *α* represents the absorption coefficient, K is a constant, E_p_ is photon energy, and E_g_’ is the band gap energy. *α* is determined by Equation (4), where A is the absorption (arbitrary units) and d is the sample thickness, which, for a cuvette, is 1 cm. The photon energy was calculated using Planck’s equation, as shown in Equation (5), where hc is 1240 eV nm and λ represents the wavelength in nm. The band gap energy, E_g,_ was determined by linear extrapolation of absorption edges on the graph of (αE_p_)^2^ versus E_p_ to the *x*-axis where y = 0. 

Density functional theory (DFT) calculations were performed using the B3LYP functional, which is composed of the Becke 88 exchange functional [15] in combination with the LYP correlation functional [16], as implemented in the Gaussian 16 package [17]. The triple-ζ basis set 6-311G(d,p) was used for lighter atoms (C, H, N, O) and the def2tzv basis set for both the core and valence electrons of Zr. Optimisations and time-dependent DFT (TDDFT) calculations were performed in the gas phase. The input coordinates for the ligands were constructed using Chemcraft [18]. The input coordinates for the model Zr-AZB-molecule was constructed in Chemcraft using published coordinates from the literature [12]. Visualisations of optimised molecules were performed using Chemcraft [18].

## 3. Photo-Isomerisation Kinetics of AZB(COOH)_2_ Ligand

Azobenzene derivatives are photo-responsive compounds that exist as the *trans* and the *cis* isomer. The *trans* isomer exists under ambient light conditions, whereas the *cis* isomer is generated through irradiation with ultraviolet light [19]. The thermal *cis* to *trans* isomerisation, after irradiation, is proposed to proceed via in-plane inversion [20], out of plane rotation around the N=N double bond [21], or a combination of both [22], depending on the solvent polarity and the position and type of substituents. The kinetics of the thermal *cis* to *trans* isomerisation of substituted azobenzene is independent of the *cis* isomer concentration, i.e., how long the sample has been irradiated before the kinetic measurements (first-order) [20]. No data for the thermal *cis* to *trans* isomerisation of AZB(COOH)_2_, or *trans*–*cis* isomerisation under irradiation of UV light of azobenzenes, could be found in the literature.

**Figure 1:**
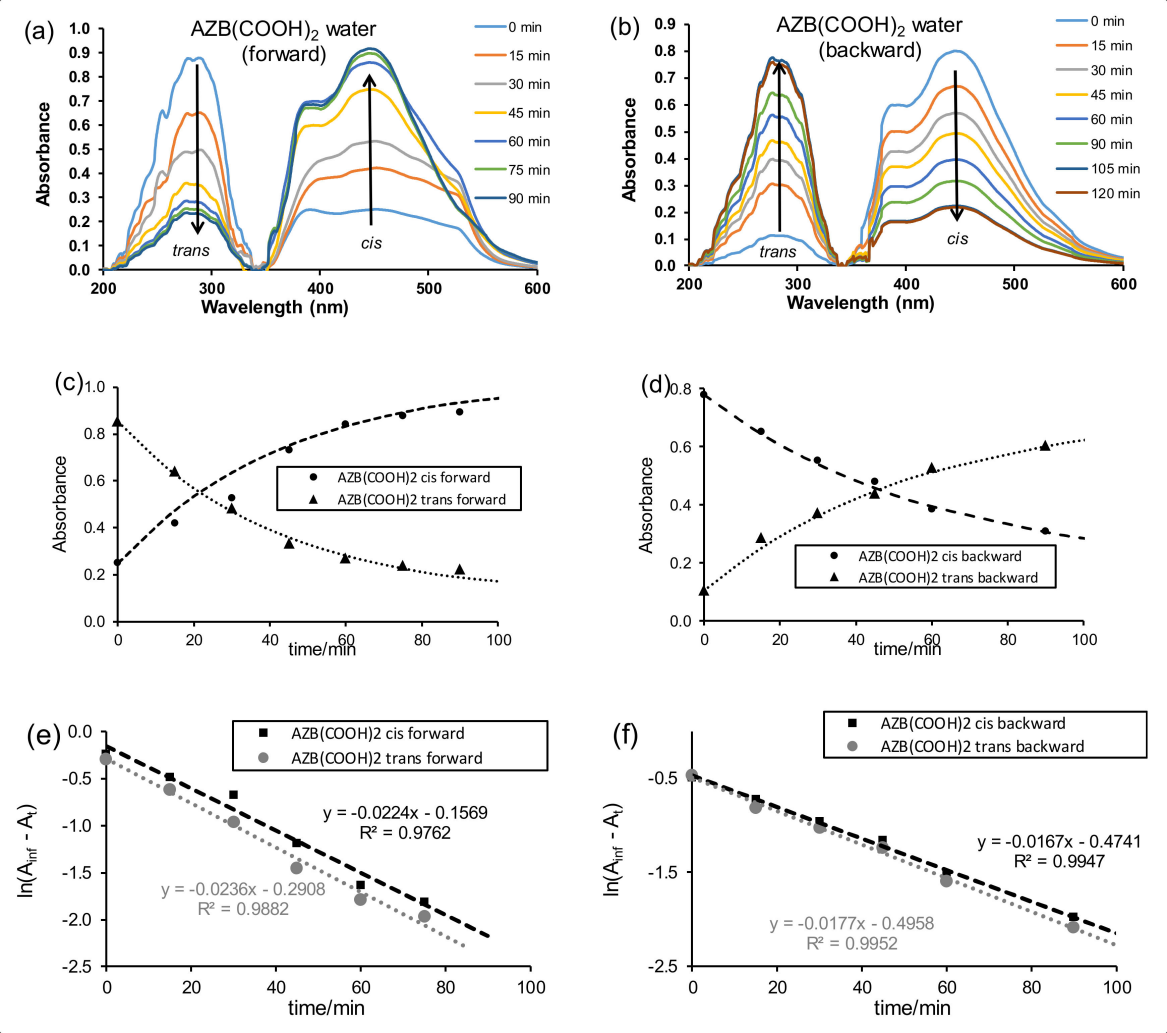
AZB(COOH)_2_ (10 ppm in water) plots of *trans*–*cis* (forward) isomerisation (**a**,**c**,**d**) and *cis–trans* (backward) isomerisation (**b**,**d**,**f**). (**a**,**b**) UV–Vis absorbance vs. wavelength, (**c**,**d**) absorbance (A) vs. time, (**e**,**f**) ln(A_inf_ - A_t_) vs. time.

The ligand AZB(COOH)_2_ was dissolved in the solvents (water, DMF, CHCl_3_) and irradiated with UV light to follow the ligand’s photo-responsive nature. Before irradiation (time t_0_), both the *cis* and *trans* isomers were present, with the *trans* isomer as the major component; see Figure 1a at time t = 0 for AZB(COOH)_2_ dissolved in water as an example. Both the *trans*–*cis* (forward) isomerisation under irradiation of UV light and the *cis–trans* (backward) isomerisation of the free AZB(COOH)_2_ ligand after irradiation with UV light for 18 h to obtain a *cis* saturated solution were followed on UV–Vis in the solvents water, DMF, and CHCl_3_. The first order isomerisation rate constants obtained are summarised in Table 1. 

**Figure 2:**
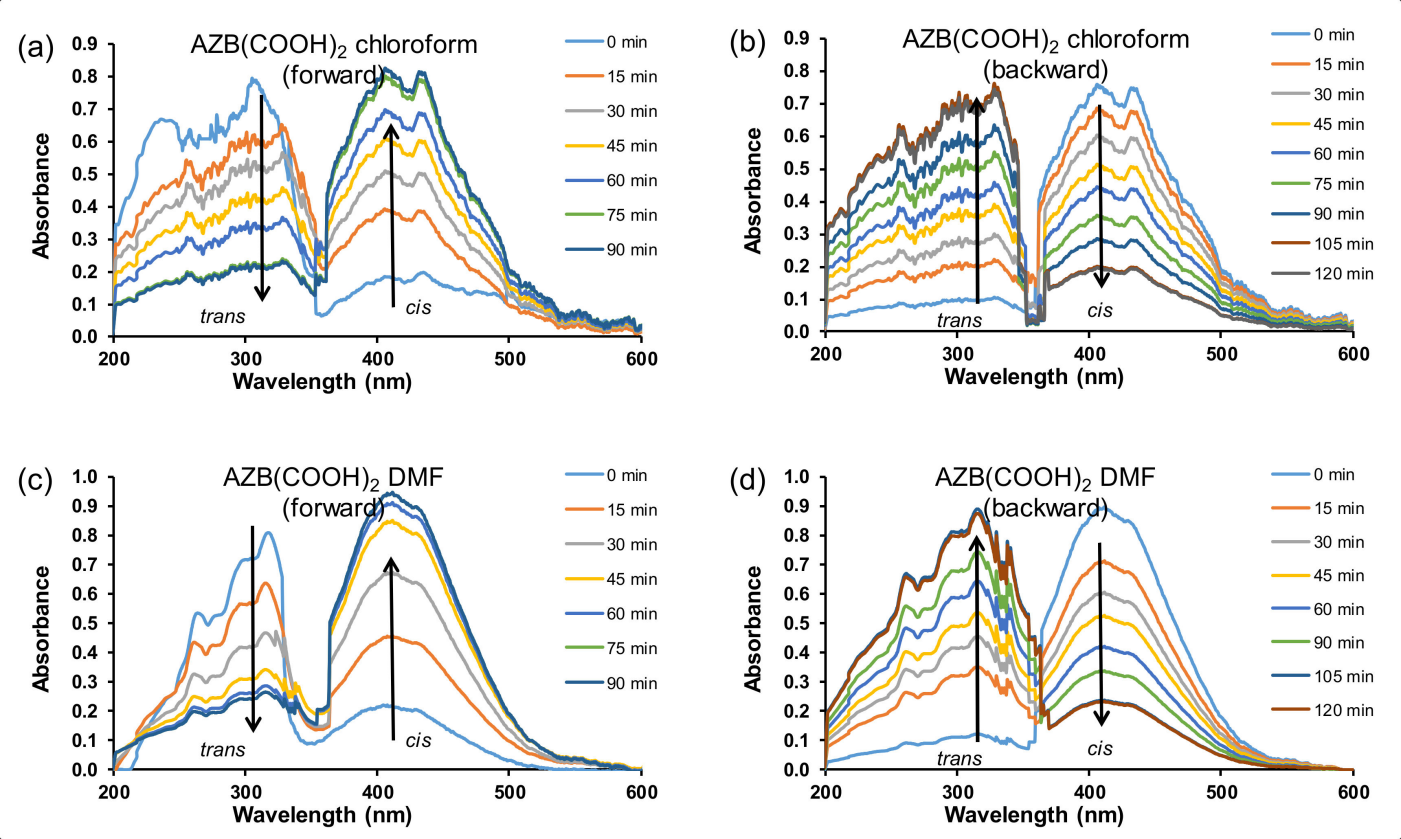
UV–Vis absorbance vs. wavelength AZB(COOH)_2_ plots of *trans*–*cis* (forward) isomerisation (**a**,**c**) and *cis–trans* (backward) isomerisation (**b**,**d**) in the indicated solvents with concentrations of 15 ppm in chloroform and 20 ppm in DMF.

The *trans–cis* isomerisation of the free AZB(COOH)_2_ ligand in water (10 ppm or 4 × 10^−5^ M) under irradiation of UV light, as followed on UV–Vis (Figure 1a,c), reached equilibrium after ca 120 min. Photo-isomerisation was observed by the decrease of the UV–Vis absorption band at 294 nm (*trans*) and an increase of the absorption band at 445 nm (*cis*). The assignment of the *trans* and *cis* absorption peaks was in accordance with the literature [22] and confirmed by a TDDFT study presented in Section 5 below. The rate constants for the *trans–cis* (forward) isomerisation obtained from absorbance data at 294 nm (0.0236 min^−1^ or 0.00039 s^−1^) and that measured at 445 nm (0.0224 min^−1^ or 0.00037 s^−1^) were kinetically the same (Figure 1e). The rate constants for the *cis–trans* (backward) isomerisation (Figure 1b,d) obtained from absorbance data at 294 nm (0.0177 min^−1^ or 0.00030 s^−1^) and that measured at 445 nm (0.0167 min^−1^ or 0.00028 s^−1^) were kinetically the same (Figure 1f). The average rate constant for the *cis–trans* (backward) isomerisation (0.017(1) min^−1^ or 0.00029(1) s^−1^) was slightly slower than the average rate constant for the *trans–cis* (forward) isomerisation (0.023(1) min^−1^ or 0.00038(1) s^−1^) for 4 × 10^−5^ M solution of AZB(COOH)_2_. Similarly, it was found that, for AZB(COOH)_2_ in (i) DMF, the *cis–trans* (backward) isomerisation (0.013(1) min^−1^ or 0.00022(1) s^−1^) was slightly slower than the average rate constant for the *trans–cis* (forward) isomerisation (0.29(6) min^−1^ or 0.0005(1) s^−1^), and in (ii) chloroform, the *cis–trans* (backward) isomerisation (0.010(1) min^−1^ or 0.00017(2) s^−1^) was slightly slower than the average rate constant for the *trans–cis* (forward) isomerisation (0.18(1) min^−1^ or 0.00030(1) s^−1^); see Figure 2 for the UV–Vis kinetic graphs. 

Reported *cis–trans* (backward) isomerisation rates in DMSO for a series of halogen-substituted azobenzenes (1 × 10^−5^ M) vary between 0.0002 and 0.0008 s^−1^ [1], similar to the values obtained here for the isomerisation of AZB(COOH)_2_ of 0.00029(1) s^−1^ in water (4 × 10^−5^ M), 0.00022(1) s^−1^ in DMF (7 × 10^−5^ M), and 0.00017(2) s^−1^ in chloroform (6 × 10^−5^ M). However, the reported *cis–trans* isomerisation rate for unfunctionalised AZB, AZB(H)_2_ (2.6 × 10^−5^ M), in water is much slower 0.00000017(3) s^−1^ [22].

## 4. Photo-Isomerisation Kinetics of MOFs

**Figure 3:**
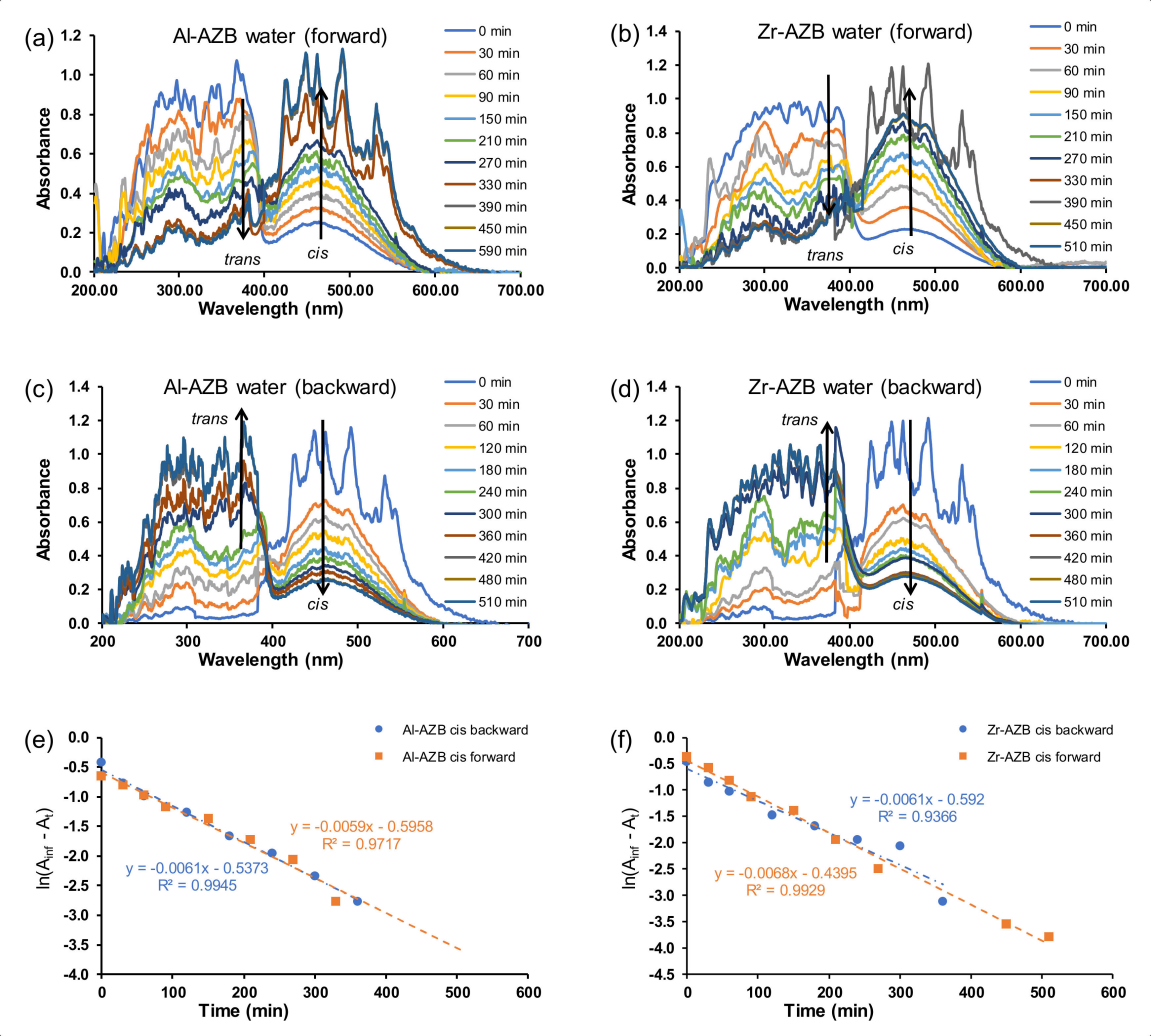
Plot for absorbance vs. wavelength for the forward (*trans–cis*) isomerisation of (50 ppm) (**a**) Al-AZB and (**b**) Zr-AZB. Plot of absorbance vs. wavelength for the backward (*cis–trans*) isomerisation (**c**) Al-AZB and (**d**) Zr-AZB. Plot of ln(A_inf_ - A_t_) vs. time for (**e**) Al-AZB and (**f**) Zr-AZB in water.

**Figure 4:**
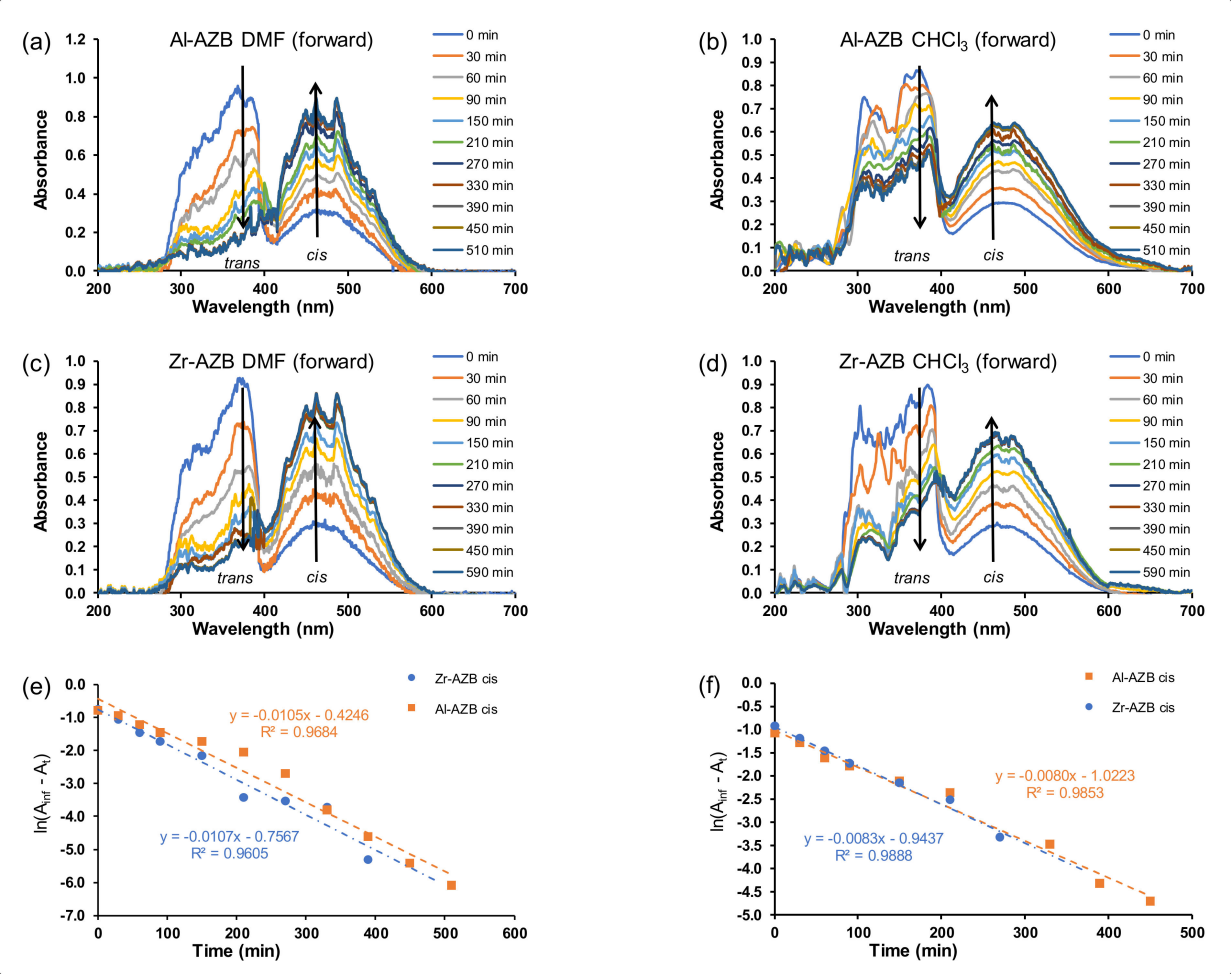
Plot of absorbance vs. wavelength for the forward (*trans–cis*) isomerisation in DMF (50 ppm) for (**a**) Al-AZB and (**c**) Zr-AZB. Plot of absorbance vs. wavelength in CHCl_3_ (50 ppm) for (**b**) Al-AZB and (**d**) Zr-AZB. Plot of ln(A_inf_ - A_t_) vs. time for Al-AZB and Zr-AZB in (**e**) DMF and (**f**) CHCl_3_.

After the AZB(COOH)_2_ ligands were built into the MOF structures (Al-AZB and Zr-AZB), the UV–Vis spectra of the MOF samples suspended in the solvents (water, DMF, CHCl_3_), showed that mainly the *trans* but also the *cis* isomer were present in the suspensions. The suspended Al-AZB and Zr-AZB samples were irradiated with UV light to follow the MOFs’ photo-responsive nature. The forward *trans-*to-*cis* (Figure 3a,b and Figure 4a–d) and backward *cis*-to-*trans* (Figure 3c,d) isomerisation of the azobenzene ligand on the MOFs were evaluated. The kinetics were followed by the change of absorbance at 457 nm since the curve at 380 nm was jagged. The rate of azobenzene isomerisation in both Al-AZB and Zr-AZB is ca three - four times slower than that for the free ligand in water (Figure 3e,f; Table 1). The ligands did not detach during the kinetic experiments since the observed absorbance–wavelength data did not correspond with the absorbance–wavelength spectrum of the free ligand (compare the spectra in chloroform of the free ligand in Figure 2a with the spectrum of Al-AZB in Figure 4b and that of Zr-AZB in Figure 4d in chloroform, for example). During MOF synthesis, the ligand is incorporated into the MOF backbone in its thermodynamically favoured *trans* conformation, and because of steric hindrance, the incorporated ligand encounters difficulty photo-switching to the *cis* isomer. Therefore, the observed switching is attributed to partially solvated, singly bound ligands on the MOFs [7,23]. PXRD patterns of the MOFs taken after suspension and irradiation showed no change in the crystallinity and topology of the MOFs, an indication that photo-switching did not occur in the bulk material (SI Figures S4 and S5). These singly bound ligands do not make the material insufficient and are inherent to the MOF’s structure. Similarly, a decrease in the isomerisation rate relative to the free azobenzene was observed with covalently-bound azobenzene moieties on a graphene support [8,9]. 

After being irradiated with UV light overnight, the MOFs were exposed to ambient light, and the *cis* isomer was switched back to the *trans* conformation (Figure 3c,d). The backward isomerisation (*cis*-to-*trans*) in water for both Al-AZB and Zr-AZB showed no significant difference in the rate of the forward and backward isomerisation.

In addition to side groups attached to the benzene rings and solid supports, the rate of isomerisation of azobenzene derivatives is also dependent on the solvent used to study the isomerisation [1,2,24,25]. The difference in the isomerisation rate constants is correlated to solvent effects, such as polarity [2,25,26]. Typically, in polar solvents, azo-compounds form hydrogen bonds with the nitrogen double bond to form a hydrazone-like electronic distribution. The breaking of the nitrogen double bond by hydrogen bonding causes faster isomerisation around the nitrogen atoms [2]. The isomerisation during irradiation over 8 h was further investigated in dimethylformamide and chloroform (Figure 4). The rate of isomerisation in the different solvents in this study slightly increased in the following order: water (1.0 × 10^−4^ s^−1^) < chloroform (1.4 × 10^−4^ s^−1^) in DMF (1.8 × 10^−4^ s^−1^). The plots for absorbance vs. time in the different solvents is given in the supplementary information (Figures S6 and S7).

In our study, there was not a significant difference in the rate of isomerisation between the aluminium and zirconium MOFs, although there was a markable difference between the free ligand and the MOFs. Although there are no reports on the kinetic rate constants of azobenzene MOFs, there are reports of azobenzenes covalently bound to a graphene solid support. Therefore, Table 1 compares the kinetic rate constants for free azobenzene derivative ligands, as well as those covalently bonded to a graphene solid support. *Trans*–*cis* isomerisation kinetic rate constants were orders of magnitude smaller when covalently bonded to a graphene solid support compared to the free azobenzene.

## 5. DFT Study 

**Figure 5:**
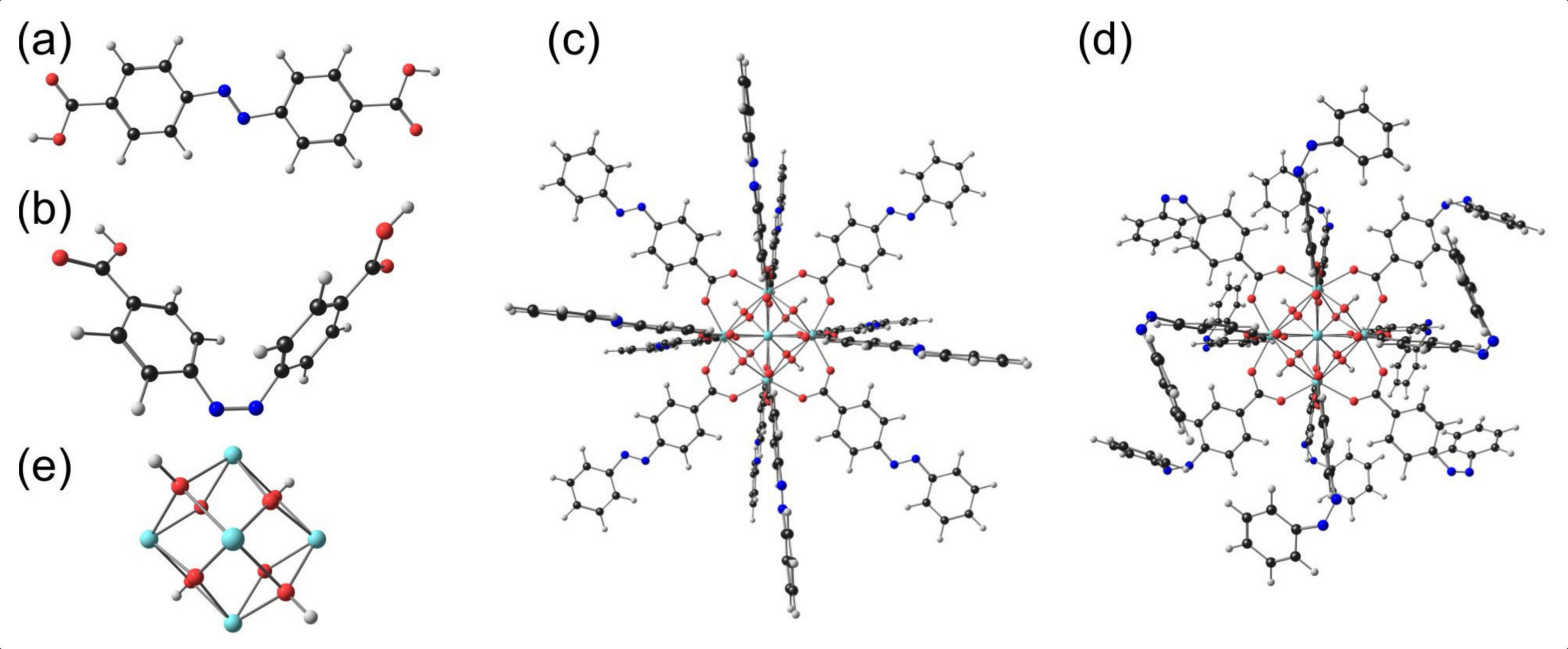
B3LYP gas phase optimised (**a**) *trans* and (**b**) *cis* AZB(COOH)_2_. A model molecule of Zr-AZB: (**c**) *trans* model-Zr-AZB and (**d**) *cis* model-Zr-AZB. For simplification, the terminal COOH groups in the singly bound ligands were replaced by H. (**e**) The central Zr_6_H_4_O_8_ unit of the Zr-AZB MOF (enlarged).

For further insight into the structure and observed UV–Vis spectra of the AZB(COOH)_2_ ligand and the MOFs, we optimised both the *trans* and *cis* AZB(COOH)_2_ ligand molecules, as well as a small model molecule with the same coordination configuration as Zr-AZB, model-Zr-AZB. The optimised molecules are shown in Figure 5. Zr-AZB MOFs consist of central Zr_6_H_4_O_8_ units (Figure 5c), with each unit connected to 12 AZB(COO)_2_ linkers. Each Zr of the central Zr_6_H_4_O_8_ unit is 8-coordinated and connected to four bridged O between the Zr atoms in the central unit and to one O of four different AZB(COO)_2_ linkers. Each experimental Zr-AZB MOF crystal consists of many of these units connected to each other through the linkers. Because of the inhered structure of the Zr-AZB MOF, the surface of Zr-AZB will necessarily contain singly bound ligands that can be in the *trans* or *cis* conformation. Model-Zr-AZB consists only of one central Zr_6_H_4_O_8_ unit with 12 AZB(COO)_2_ linkers with the terminal COOH groups in the singly bound ligands replaced by H for simplification. The DFT optimised structures of *trans* model-Zr-AZB and *cis* model-Zr-AZB, with the singly bound linkers on the surface optimised in the *trans* and *cis* conformations, respectively, are shown in Figure 5c,d. Note that the surface of the experimental Zr-AZB MOF contains a mixture of singly bound *trans* and *cis* ligands.

**Figure 6:**
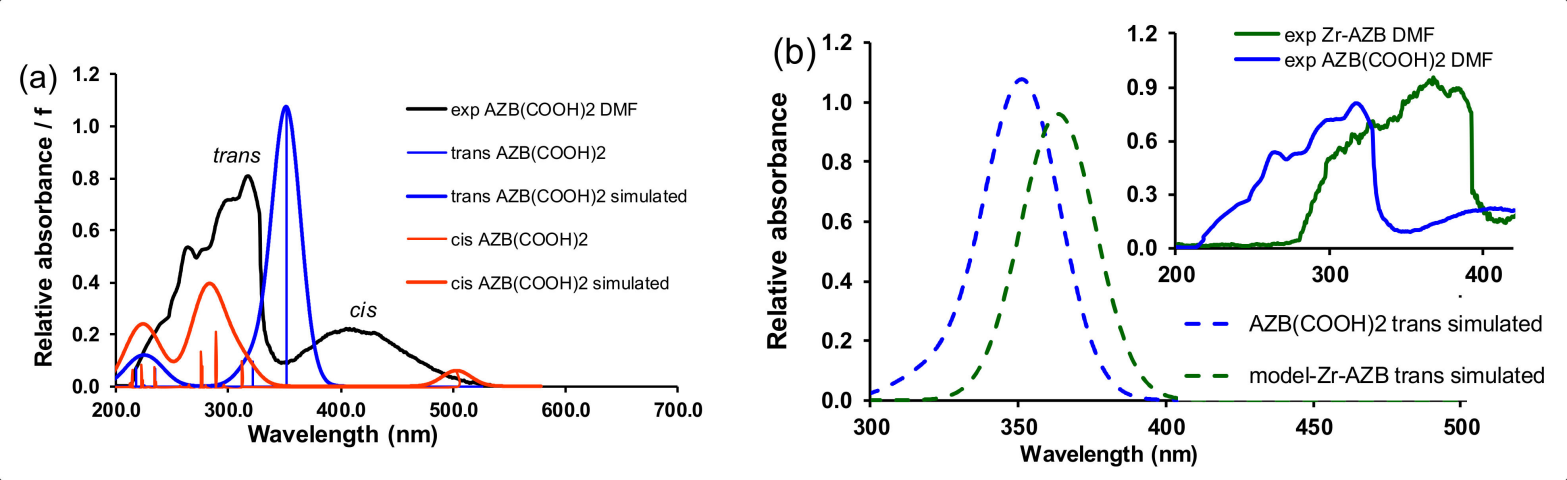
B3LYP gas phase TDDFT calculated oscillators and Gaussian broadening curves (30 nm halfwidth) of (**a**) *trans* (blue curve) and *cis* (red curve) AZB(COOH)_2_ with the experimental spectrum in DMF shown in black, (**b**) *trans* (blue curve) AZB(COOH)_2_ and *trans* model-Zr-AZB (green curve) with the corresponding experimental spectra in DMF shown in the inset in blue (*trans* peak of AZB(COOH)_2_) and green (*trans* peak of Zr-AZB).

TDDFT calculations (Figure 6a) show a redshift of ca 60 nm for the calculated UV–Vis spectrum of the *cis* AZB(COOH)_2_ compared to the *trans* AZB(COOH)_2_, in qualitative agreement with the experimental spectra containing a mixture of *trans* and *cis* AZB(COOH)_2_ (Figure 1 and Figure 2). The gas phase calculated UV/Vis spectrum of *trans* model-Zr-AZB exhibits a redshift of 41 nm compared to *trans* AZB(COOH)_2_, in agreement with the trend of the experimental spectra, which shows a redshift of 73 nm in DMF; see Figure 6b. 

## 6. Half-Life

The half-life of the MOFs at 25 °C in various solvents is an indication of their stability (Table 2). The half-life of azobenzene dicarboxylic acid ligand upon irradiation with UV light (*trans*) in water (30 min) increases (65–118 min) upon coordination with the metals. The *trans* isomer bound to the MOFs is thus more stable than the free ligand. The singly bound *trans* isomers incorporated into the MOF’ backbone encounter difficulty photo-switching to the *cis* isomer because of the structural constraints of the crystal lattice. The coordination of metal centres to the ligand, therefore, slows down the rate of photo-isomerisation, which ultimately enhances the stability of the kinetically favoured *trans* isomer. While the half-life of the *trans*–*cis* isomerisation of AZB, Al-AZB, and Zr-AZB are measured in minutes, the half-life of the *cis*–*trans* isomerisation of azobenzenes covalently bound onto a graphene solid support are 5–225 days. Table 2 compares the half-life of azobenzene-based MOFs with that of azobenzene-bound graphene.

## 7. Tauc Plot

**Figure 7:**
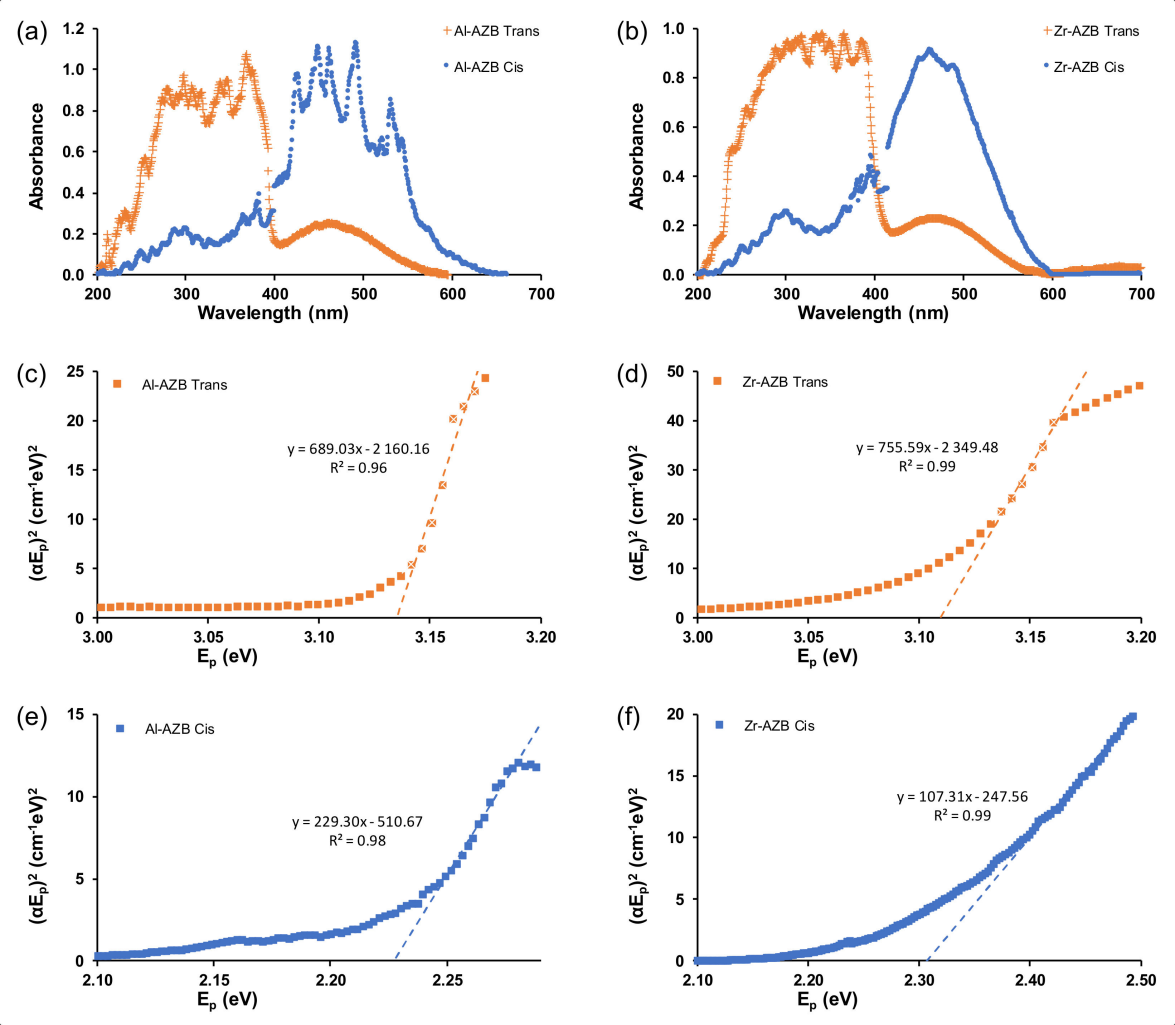
The UV–Vis spectra in water of *trans* and *cis* forms of (**a**) Al-AZB and (**b**) Zr-AZB as well as Tauc plots of (**c**) Al-AZB *trans* isomer, (**d**) Zr-AZB *trans*, (**e**) Al-AZB *cis*, and (**f**) Zr-AZB *cis*.

To further understand the energy efficiency of the MOFs, their band gap energies were obtained from the Tauc plots of the isomers in the different solvents, shown in Figure 7 and Figures S8–S10 of the supplementary information. For MOFs, the band gap energy can be modified by altering the organic linker or metal ion [29]. Much research is being conducted on the reduction of the band gap energies of inorganic semi-conductors, as materials with lower band gap energies are often more energy-efficient in photocatalysis [30].

The band gap energies of both Al-AZB and Zr-AZB were similar at 3.1 eV for the *trans* isomers and ca 2.2 for the *cis* isomers. Al-AZB and Zr-AZB are thus equally energy-efficient, although zirconium metal is more expensive than aluminium. The significant difference between the band gap energies of the *cis* and *trans* isomers can be exploited in many applications, such as catalysis. The *cis* isomer has a lower band gap energy than the *trans* isomer; thus, making the *cis* isomer more energy-efficient. For a series of azobenzene derivatives grafted onto a solid support [27] the *cis* isomer had a lower band gap energy than the *trans* isomer, similar to our experimental and DFT-calculated results (Table 3). Additionally, the difference in solvents did not affect the band gap significantly. However, the band gaps of the MOFs were noticeable lower than the band gap of the free AZB(COOH)_2_ ligand (*trans*: 3.88 (3) eV, *cis*:2.40 (5) eV, Supplementary Materials Figure S10). 

## 8. Fluorescence 

**Figure 8:**
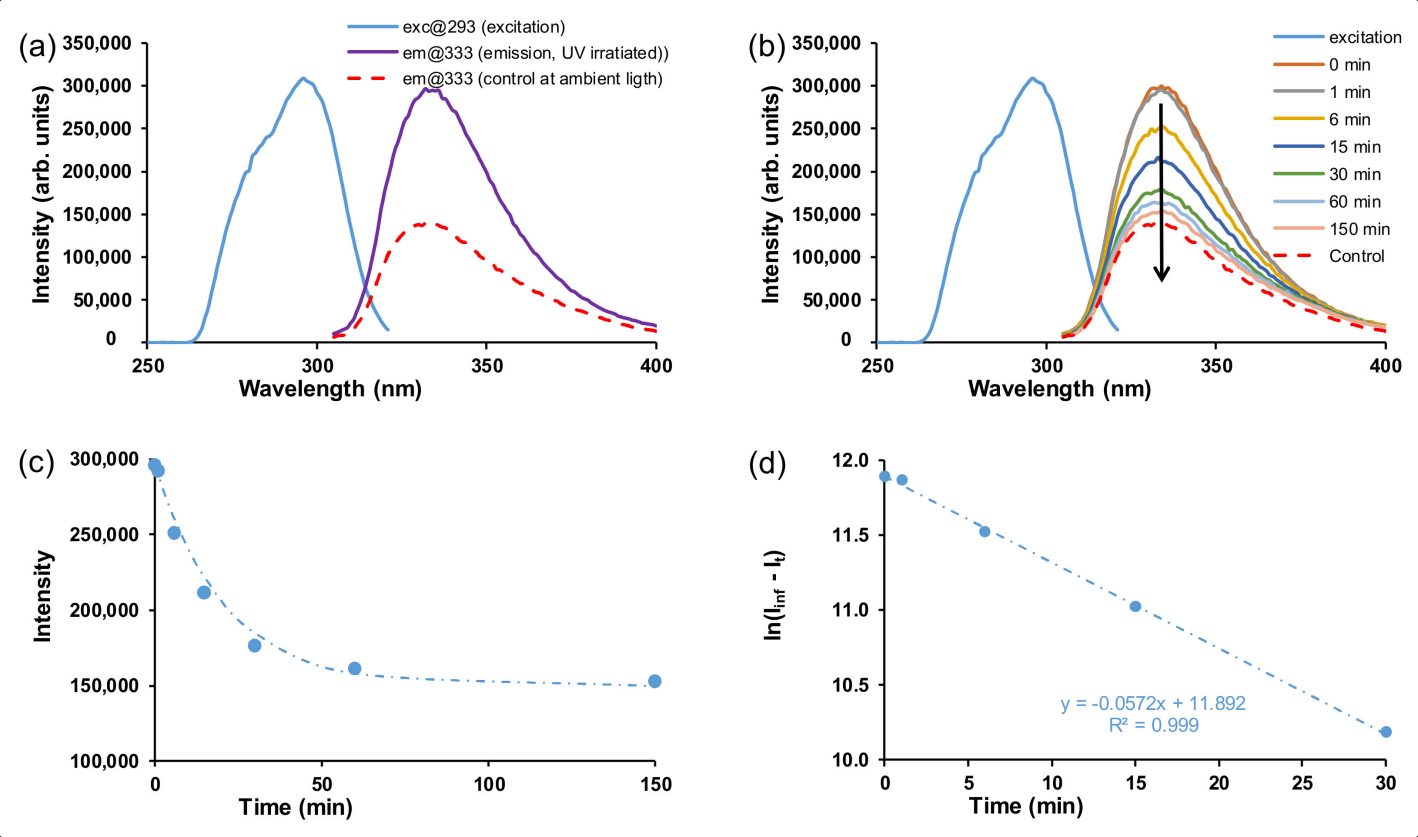
(**a**) Fluorescence spectra of the 10 ppm AZB(COOH)_2_ ligand. The excitation curves for both *trans* and *cis* overlapped at 293 nm and their emission curves are shown at 333 nm. (**b**) UV-irradiation followed by excitation and decay of the excitation curve under ambient light. (**c**) Intensity (I) vs. time and (**d**) ln(I_inf_ − I_t_) vs. time.

In general, azobenzenes are incapable of emitting fluorescence with a significant quantum yield upon UV light irradiation, despite their intense absorbance in the UV region [33]. The excited state decay of the *cis* and *trans* isomers of azobenzenes in DMSO are generally in the ps timescale [34]. It was, however, found that bulky substituents close to the N=N linker or intramolecular hydrogen bonding slowed down the rotation around the nitrogen–carbon bond during photo-isomerisation, increasing the fluorescence performance of azobenzene compounds in solution [35]. In this study, both the free ligand and crystalline MOFs did not display fluorescent behaviour in solid-state; hence, the samples were suspended in DMF. When suspended in DMF, it was found that the AZB(COOH)_2_ ligand has fluorescent properties and that the excited state of *cis*-rich AZB(COOH)_2_ in DMF decays in ca 3 h (Figure 8). Two samples of the AZB(COOH)_2_ ligand were suspended in DMF. The one sample was irradiated by UV light for 18 h while stirring to induce the *cis* conformation. The other sample (the control, mainly the *trans* conformation) was stirred under normal office light. Both *trans* and *cis* isomers were excited at 293 nm, and both were fluoresced at 333 nm (Figure 8a). The fluorescence intensity of the control (*trans*) sample (shown as em@333, control at ambient light) was half that of the excitation intensity (shown as exc@293). The emission intensity of the UV-exposed *cis* sample (shown as em@333, emission, UV-irradiated) was similar to the intensity of the excitation curve and thus has a higher emission intensity than the *trans* isomer (control) that was excited with the same excitation intensity. 

The reverse isomerisation of the UV-irradiated *cis* isomer back to *trans* was followed (Figure 8b–d), giving an isomerisation rate constant in DMF of 0.0475 min^−1^ (0.00079 s^−1^), ca 4 times higher than was observed in DMF (0.00022 s^−1^) under irradiation with UV light (Table 1). When exposed to ambient light, the *cis* isomer decayed to the same emission intensity as the control (*trans*) sample after about 150 min (2.5 h). However, upon the coordination of AZB(COOH)_2_ into the crystalline MOF structures, fluorescence was lost. It was previously shown by Pamei et al. that the coordination of certain metals reduces photoluminescence properties of the stilbene derivative linkers (structurally related to azobenzene) in MOFs [26]. Luminescent properties are sensitive to structural and coordination geometry. When coordinated with the MOFs, the variation in the structure and geometry of the azobenzene may potentially affect the photoluminescence properties of the resulting MOFs. 

## 9. Dye Adsorption

**Figure 9:**
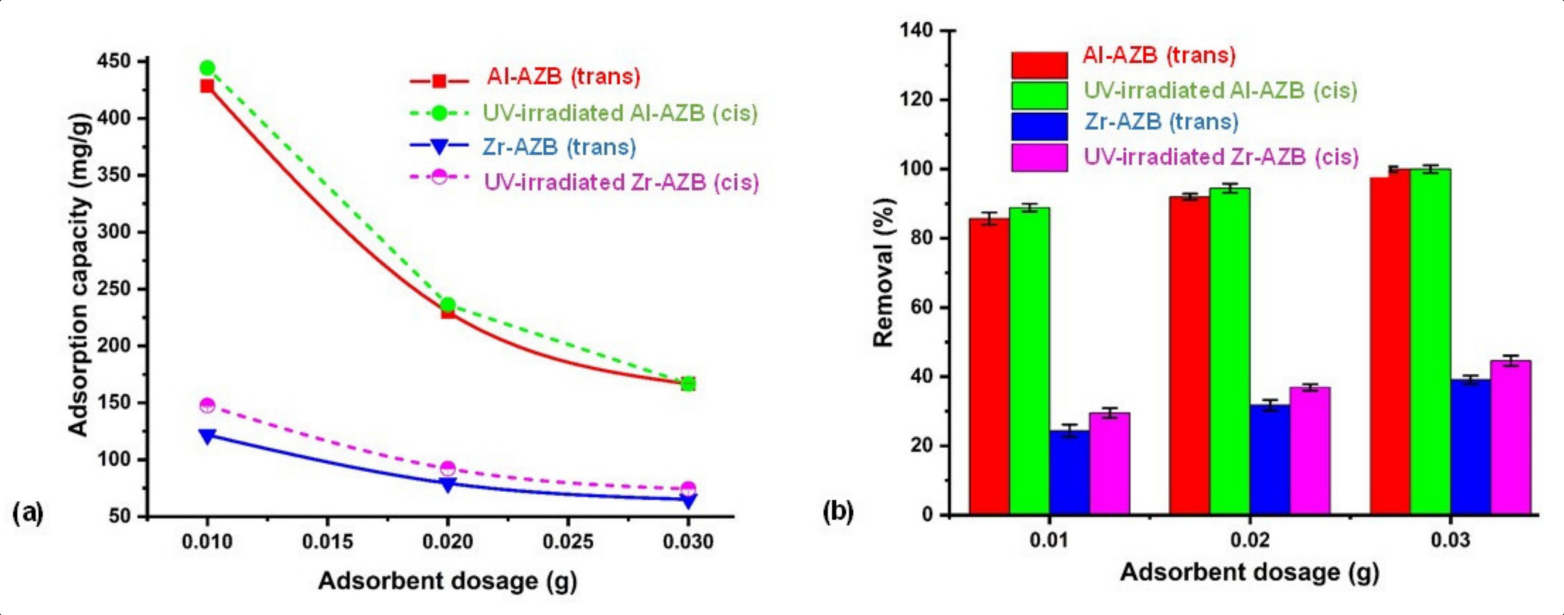
The effect of UV irradiation on the (**a**) adsorption capacity and (**b**) percentage removal of the metal organic frameworks at varying sorbent doses (pH 4.0, time 1440 min, temperature 300 K, dye concentration 500 mg/L).

In our previous study, the Zr-AZB and Al-AZB MOFs in their *trans* conformation were comprehensively tested for Congo Red (CR) dye adsorption [7]. Herein, a preliminary study on the effect of the different conformations (*cis* versus *trans*) on dye adsorption is presented in order to observe whether dye adsorption can be enhanced by photo-responsive MOFs. To obtain the *cis* samples, the MOFs were irradiated (at 365 nm) for 10 h, after which the absorption tests were performed in the dark. The comparable CR dye removal by Al-AZB and Zr-AZB in the *trans* and the *cis* conformation is reported in Figure 9. As previously reported, the more than 50% higher and more efficient adsorption of CR by Al-AZB compared to Zr-AZB is attributed to the much higher surface area (2718 m^2^/g and 1098 m^2^/g respectively), pore volume (0.86 and 0.35 cm^3^/g), and pore size (28, 30, 15 Å and 9, 16, 18 Å.) of the aluminium derivative [7]. When comparing the dye adsorption of the *cis* and *trans* conformations, it is evident that the UV-irradiated MOFs (*cis* conformation) gave a slightly higher percentage of CR removal (ca 2–6%) at various adsorbent doses (Figure 9a) compared to the non-irradiated MOFs (*trans* conformation). UV-irradiation, enabling the isomerization of both Al-AZB and Zr-AZB to the *cis*-conformations, is thus slightly more favourable for the efficient interaction with CR dye in solution than dye absorption under ambient light. This difference in adsorption between the *cis* and *trans* conformation of the MOFs is linked to the polarity of the azobenzene ligand. In the *trans* conformation, the ligand has a dipole moment of 0 D, whereas the *cis* has a dipole moment of 3.2 D [10,11]. The increased polarity of the framework enhances the interaction between the dye molecules and the frameworks, ultimately enhancing the dye uptake. Since the partial solvation of the singly bound AZB ligands occurs mainly at the external surface of the suspended MOF crystals, the enhancement effect is small but rather significant. It holds potential for the use of photo-responsive MOFs in advanced materials design.

## 10. Conclusions 

We observed the photo-responsive isomerisation of the azobenzene-containing MOFs, which was followed by UV–Vis absorption in multiple solvents. This photo-isomerisation is due to the partial solvation of singly bound ligands on the MOF’s surface when the MOFs are in suspension. The type of solvent in which photo-isomerisation occurs has a small effect on the rate constant and half-life of the MOFs. The solvent did not have a significant effect on the band gap energy. Both the *trans* and *cis* isomers of the AZB(COOH)_2_ ligand have fluorescent properties. However, upon coordination of the ligand to the metal centres in an MOF structure, fluorescence was lost. To explore the practical application of photo-responsive materials, the MOFs in the *cis* and *trans* conformations were tested for Congo Red dye adsorption. As previously reported, the Al-AZB exhibited higher and more efficient adsorption of CR than the Zr-AZB, which is attributed to the much higher surface area of the former. The current study shows that the *cis* isomers of both Al-AZB and Zr-AZB (obtained after UV irradiation of the *trans*-isomer) exhibited slightly higher adsorption of CR than the *trans* conformation.

## Figures and Tables

**Table 1:** *Cis–trans* (backward) and *trans–cis* (forward) conversion rate constants (k_obs_ in in s^−1^) obtained for the indicated compounds in different solvents in this study and literature.

Compound	H_2_O*_trans–cis_*	H_2_O*_cis–trans_*	CHCl_3*trans–cis*_	CHCl_3*cis–trans*_	DMF*_trans–cis_*	DMF*_cis–trans_*	Reference
AZB(COOH)_2_	3.8(1) × 10^−4^	2.9(1) × 10^−4^	3.0(1) × 10^−4^	1.7(1) × 10^−4^	5(1) × 10^−4^	2.2(1) × 10^−4^	this work
Al-AZB	1.0 × 10^−4^	1.0 × 10^−4^	1.3 × 10^−4^		1.8 × 10^−4^		this work
Zr-AZB	1.1 × 10^−4^	1.0 × 10^−4^	1.4 × 10^−4^		1.8 × 10^−4^		this work
AZB(H_2_)	1.7 × 10^−7^						[22]
**Compound**			**CHCl_3*cis–trans*_**		**DMF*_cis–trans_***		
AZB(H)(OH)			1.6 × 10^−3^				[2]
AZO-1 ^a^					1.7 × 10^−1^		[8]
AZO-2 ^b^					1.4 × 10^−5^		[8]
AZO-1–RGO ^c^					3.2 × 10^−5^		[8]
AZO-2–RGO ^d^					2.5 × 10^−7^		[8]
*para*-AZO ^e^					3.0 × 10^−1^		[9]
*ortho*-AZO ^f^					1.6 × 10^−6^		[9]
RGO-*para*-AZO ^g^					1.7 × 10^−6^		[9]
RGO-*ortho*-AZO ^h^					3.6 × 10^−8^		[9]

^a^ 4-((3,5-dimethoxyaniline)diazenyl)-benzene (see SI of reference [12] for structure); ^b^ 4-((3,5-dimethoxyaniline)diazenyl)−4-benzoic acid (see SI of reference [12] for structure); ^c^ 4-((3,5-dimethoxyaniline)diazenyl)-benzene covalently bound to reduced graphene oxide (RGO); ^d^ 4-((3,5-dimethoxyaniline)diazenyl)−4-benzoic acid covalently bound to RGO; ^e^ SO_3_H *para* substitution of azobenzene; ^f^ SO_3_H *ortho* substitution of azobenzene; ^g^ SO_3_H *para* substitution of azobenzene covalently bound to RGO; ^h^ SO_3_H *ortho* substitution of azobenzene covalently bound to RGO.

**Table 2:** Half-life (t_1/2_) obtained for the indicated compounds in different solvents in this study and the literature.

Compound	H_2_O*_trans–cis_*	H_2_O*_cis_*_–*trans*_	CHCl_3_	DMF	Other	Reference
AZB(H_2_)					144 min (gas phase)	[27]
					2 days (solution)	[28]
AZB(COOH)_2_	30 min	40 min	39 min ^a^	24 min ^a^		this work
Al-AZB	118 min	114 min	87 min ^a^	66 min ^a^		this work
Zr-AZB	102 min	113 min	84 min ^a^	65 min ^a^		this work
RGO-*para*-AZO				225 day		[9]
RGO-o*rtho*-AZO				116 h		[9]
AZO-2–RGO				33 day		[8]

^a^ *trans*–*cis* isomerisation.

**Table 3:** Band gap energy obtained for the indicated compounds in different solvents in this study and the literature.

	Band Gap Energy (eV)			
Compound	*Trans*	*Cis*	Medium	Reference
Zr-AZB	3.11 (26)	2.31 (4)	Water	this work
	3.13 (5)	2.27 (9)	DMF	this work
	3.14 (58)	2.22 (3)	CHCl_3_	this work
Al-AZB	3.14 (40)	2.23 (13)	Water	this work
	3.13 (40)	2.24 (6)	DMF	this work
	3.10 (12)	2.19 (1)	CHCl_3_	this work
AZB(COOH)_2_	3.88 (03)	2.40 (5)	Water	this work
AZB(H_2_)	2.8		gas phase experimental	[27,31,32]
	3.90	3.77	gas phase calculated	this work
AZB(COOH)_2_	3.65	3.56	gas phase calculated	this work

## Data Availability

The data presented in this study are available on request from the corresponding author. The data are not publicly available due to the copyright and intellectual property policies of the University of the Free State.

## Supplementary Materials

The following are available as Supplementary Information online: FTIR spectra, PXRD patterns, Pore-size distribution, SEM images, N_2_ and CO_2_ adsorption isotherms, UV-vis Absorbance plots and Tauc plots.
