# Supplementary material for: Trans–Cis Kinetic Study of Azobenzene-4,4′-dicarboxylic Acid and Aluminium and Zirconium Based Azobenzene-4,4′-dicarboxylate MOFs
Source: Molecules. 2022 Feb 17;27(4):1370. doi: 10.3390/molecules27041370 (PMC8875176; doi:10.3390/molecules27041370)
Supplement: Supplementary file 1 [file molecules-27-01370-s001.zip › molecules-1539273-supplementary.pdf]

Trans–cis Kinetic Study of Azobenzene-4,4′-dicarboxylic Acid and Aluminium and Zirconium Based Azobenzene-4,4′-dicarboxylate MOFs

Refilwe Mogale, Jeanet Conradie and Ernst H. G. Langner*

[1] Chemistry Department, University of the Free State, Bloemfontein, 9300 South Africa
* Correspondence: *langneeh@ufs.ac.za (E. H G. Langner)

Contents

Characterization 2
FTIR and thermal analysis 2
PXRD and Pore size distribution 3
Microscopy and surface area 4
ASAP and PXRD after 3 hour suspension and irradiation 5
Photokinetics 7
Tauc plots 8
References 10

Characterization

Al-AZB and Zr-AZB were characterized for textural and surface as reported and discussed in our previous work [1]. SEM samples were examined with a JSM-7800F-Extreme-resolution analytical field emission instrument. Powdered X-ray diffraction (PXRD) analyses were conducted on a Bruker AXS D8 Advance. The Micromeritics ASAP 2020 and ASAP 2020 v2.0 software were used to obtain porosity and surface area data. Data refinement was carried out with Microactive v1.01. For Thermo-gravimetric analyses (TGA), was performed on the Mettler-Toledo TGA/SDTA851 under a nitrogen atmosphere. The thermograms were analysed by STAR SW 8.10 software.

FTIR and thermal analysis

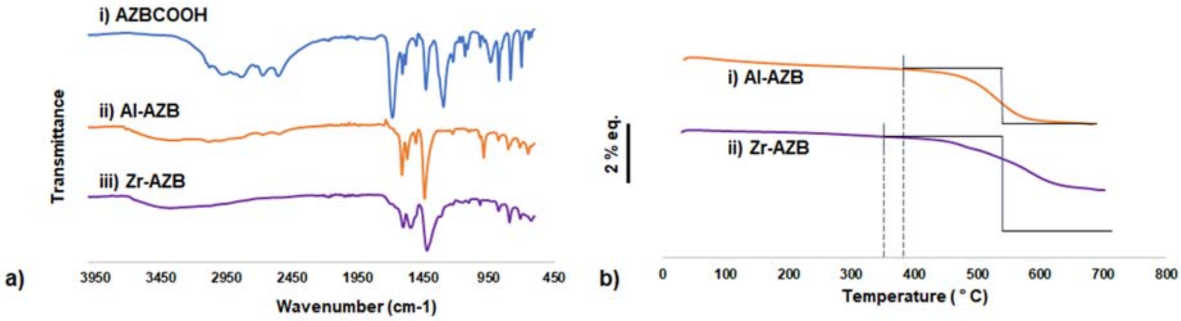

Figure S 1. (a) FTIR spectra of (i) $AZB(COOH)_2$, (ii) Al-AZB, (iii) Zr-AZB and (b) TGA thermograms of the AZB MOFs. Reprinted from Journal of Environmental Management, 304, Refilwe Mogale, Kovo G. Akpomie, Jeanet Conradie, Ernst H.G. Langner, Dye adsorption of aluminium- and zirconium-based metal organicframeworks with azobenzene dicarboxylate linkers, 114166, Copyright (2021), with permission from Elsevier.

PXRD and Pore size distribution

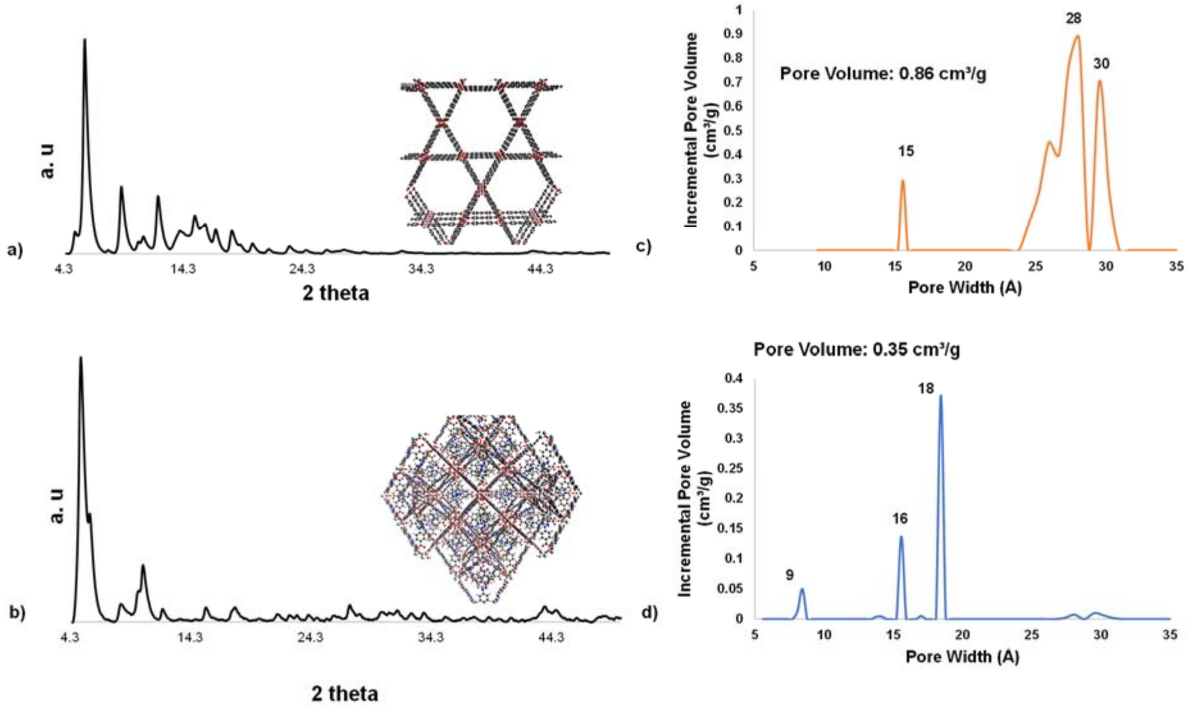

Figure S 2. The PXRD diffractograms of (a) Al-AZB and (b) Zr-AZB (bottom) alongside the DFT pore size distribution of (c) Al-AZB and (d) Zr-AZB. Reprinted from Journal of Environmental Management, 304, Refilwe Mogale, Kovo G. Akpomie, Jeanet Conradie, Ernst H.G. Langner, Dye adsorption of aluminium- and zirconium-based metal organicframeworks with azobenzene dicarboxylate linkers, 114166, Copyright (2021), with permission from Elsevier.

Microscopy and surface area

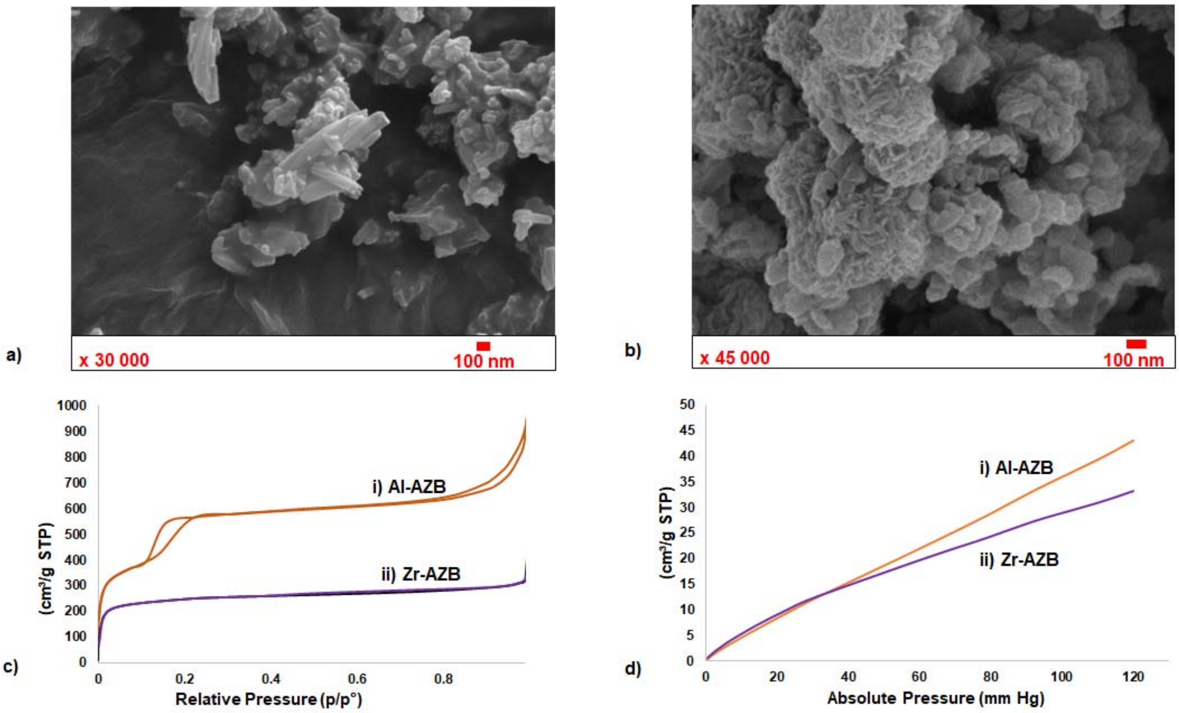

Figure S 3. SEM images of (a) Al-AZB and (b) Zr-AZB and (c) Nitrogen (N_2) at 77 K and (d) carbon dioxide (CO_2) at 273 K isotherms of (i) Al-AZB and (ii) Zr-AZB. Reprinted from Journal of Environmental Management, 304, Refilwe Mogale, Kovo G. Akpomie, Jeanet Conradie, Ernst H.G. Langner, Dye adsorption of aluminium- and zirconium-based metal organicframeworks with azobenzene dicarboxylate linkers, 114166, Copyright (2021), with permission from Elsevier.

ASAP and PXRD after 3 hour suspension and irradiation

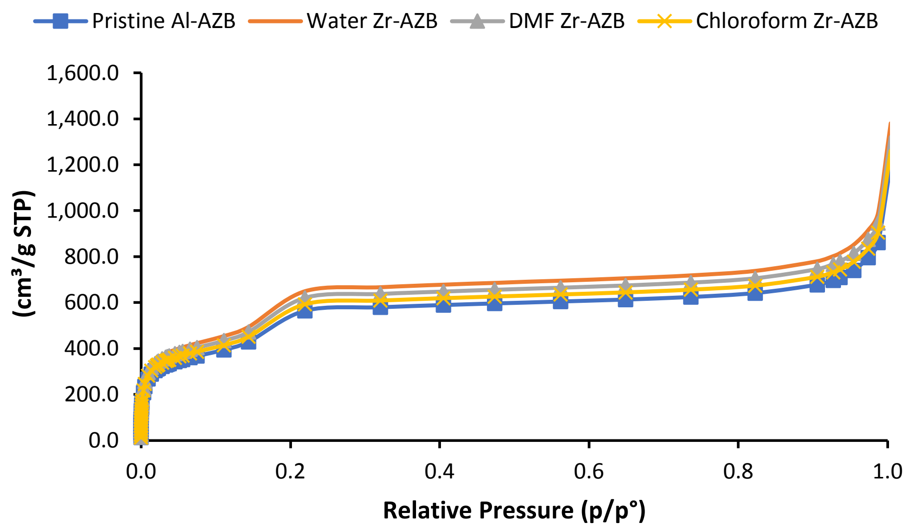

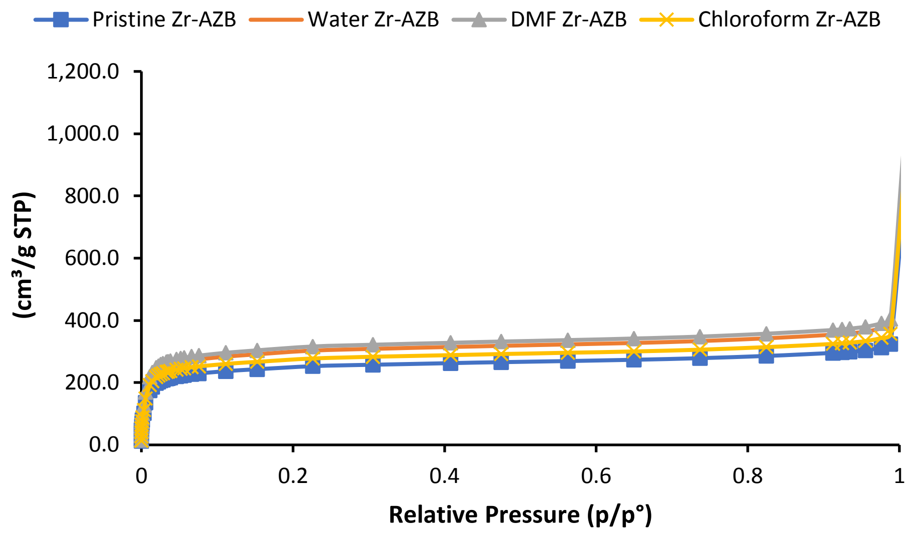

Figure S 4. ASAP after 3 hour suspension and irradiation.

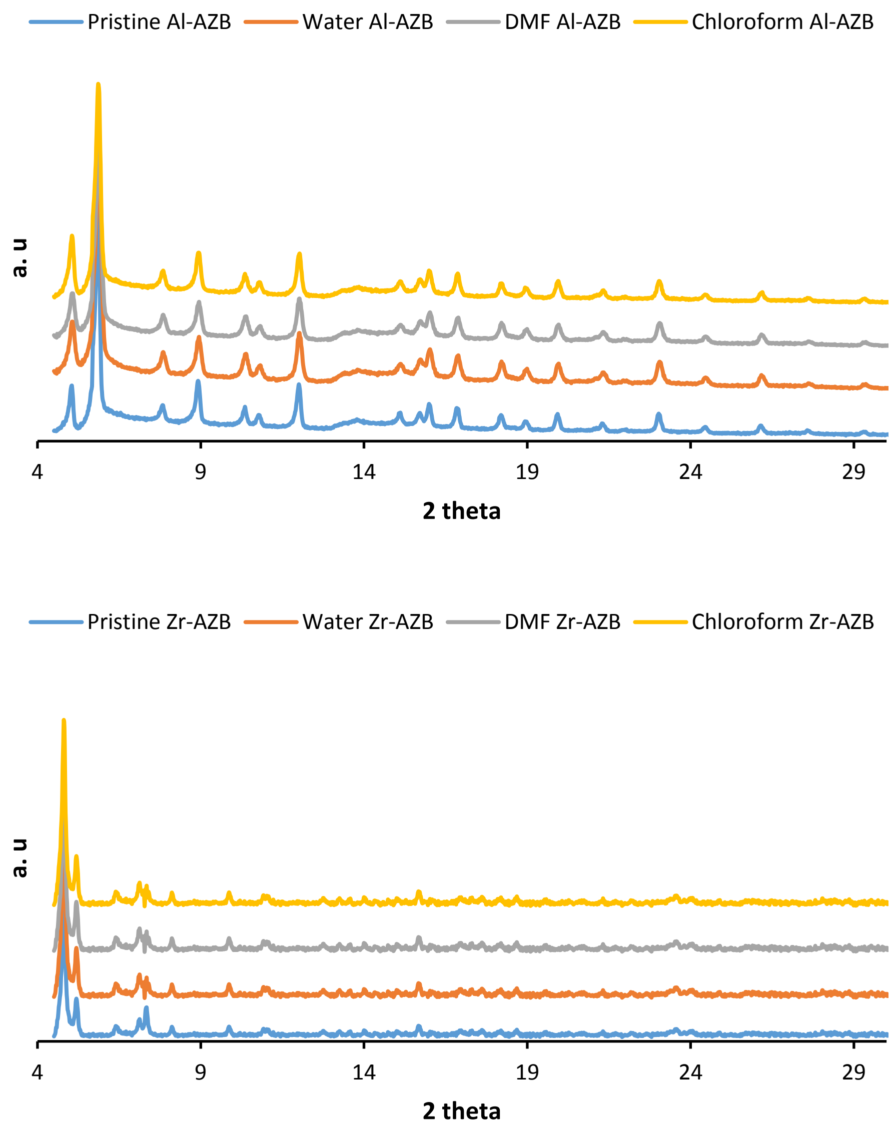

Figure S 5. PXRD after 3 hour suspension and irradiation.

Photokinetics

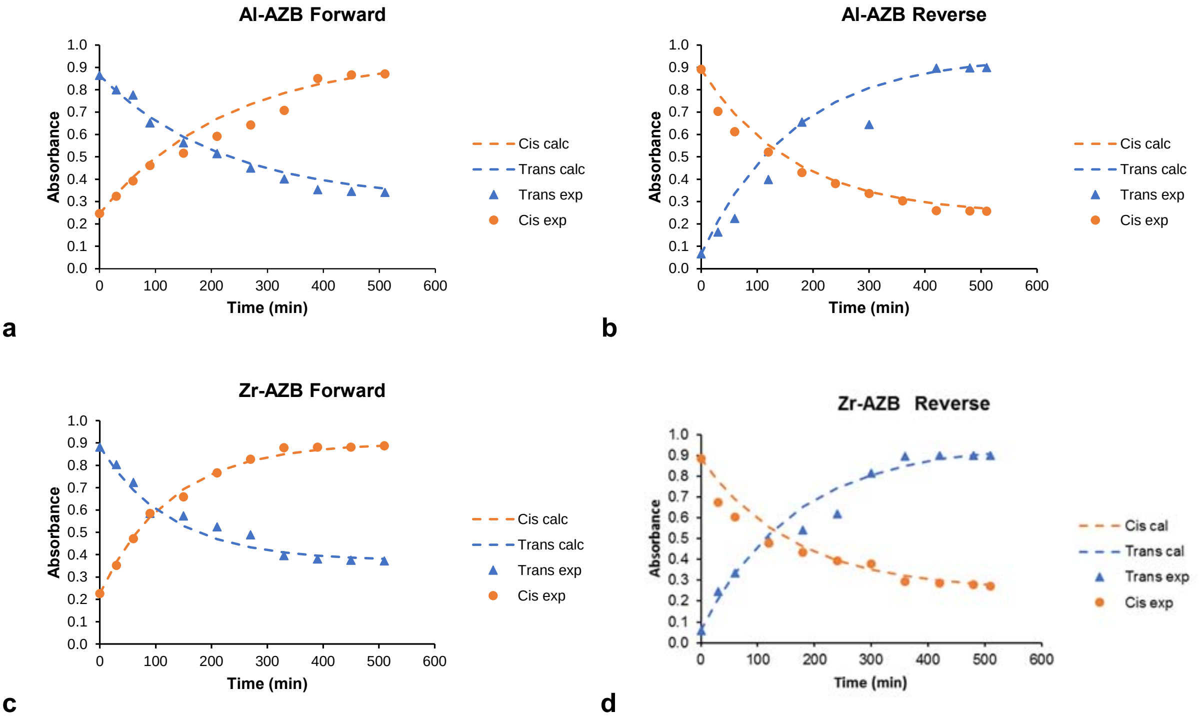

Figure S 6. The plot for absorbance *vs* time for **a)** Al-AZB for the forward isomerization **b)** Al-AZB for the reverse (backward) isomerization **c)** Zr-AZB for the forward isomerization and **d)** Zr-AZB for the reverse isomerization in water.

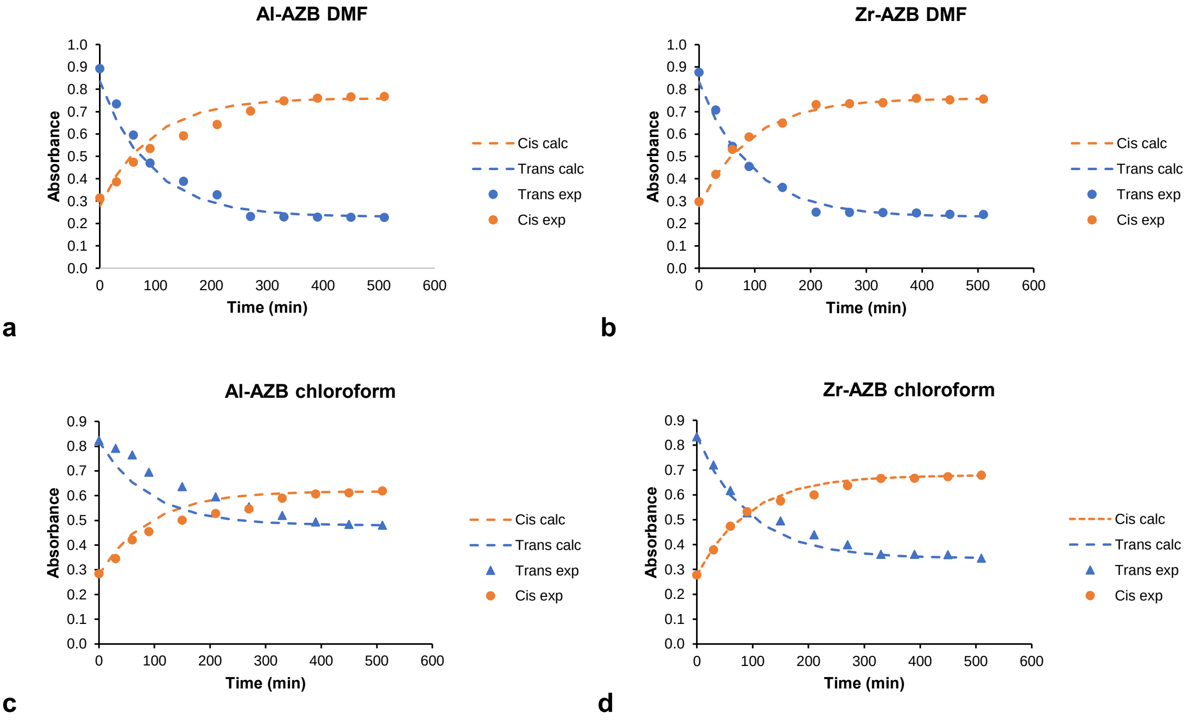

Figure S 7. The plot for absorbance *vs* time for the forward isomerization of Al-AZB in **a)** DMF and **b)** $CHCl_3$. As well as Zr-AZB in **c)** DMF and **d)** $CHCl_3$.

Tauc plots

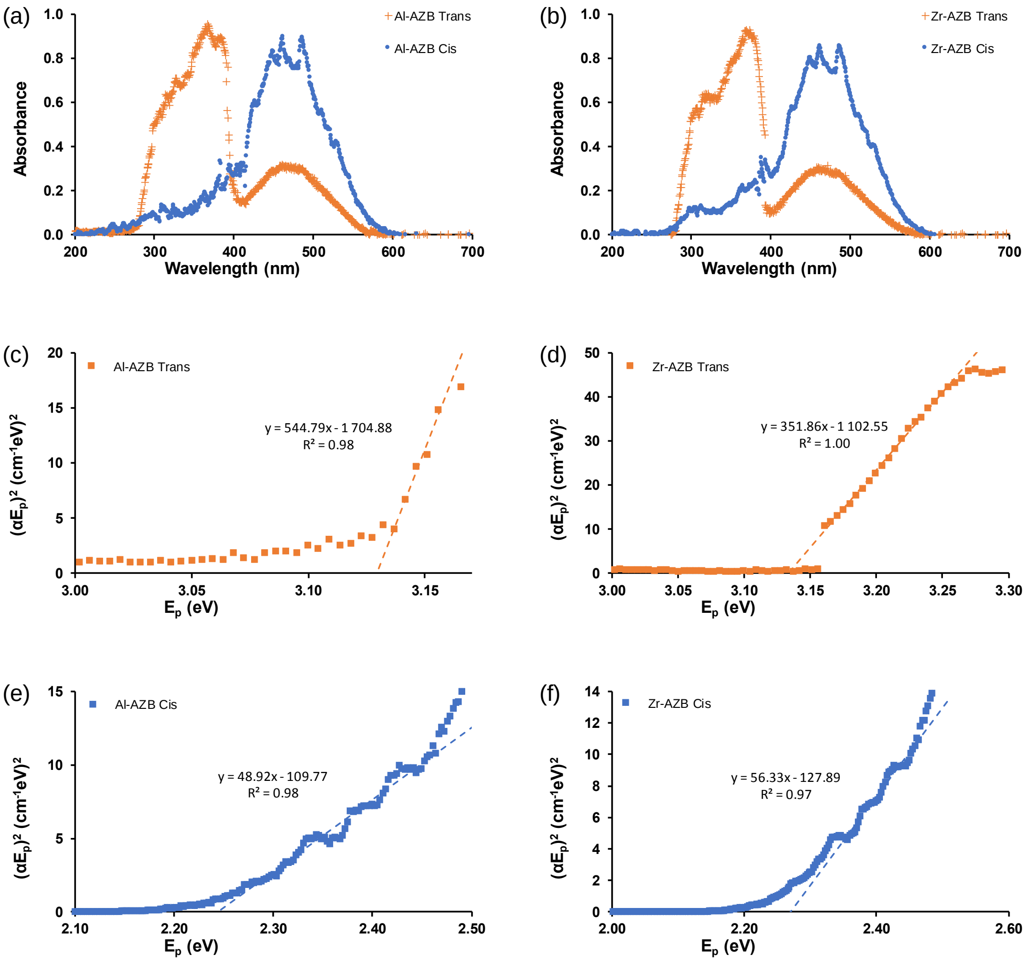

Figure S 8. The UV-Vis spectra in DMF of *trans* and *cis* form of **a)** Al-AZB and **b)** Zr-AZB as well as Tauc plots of **c)** Al-AZB *trans* isomer, **d)** Zr-AZB *trans*, **e)** Al-AZB *cis* and **f)** Zr-AZB *cis*.

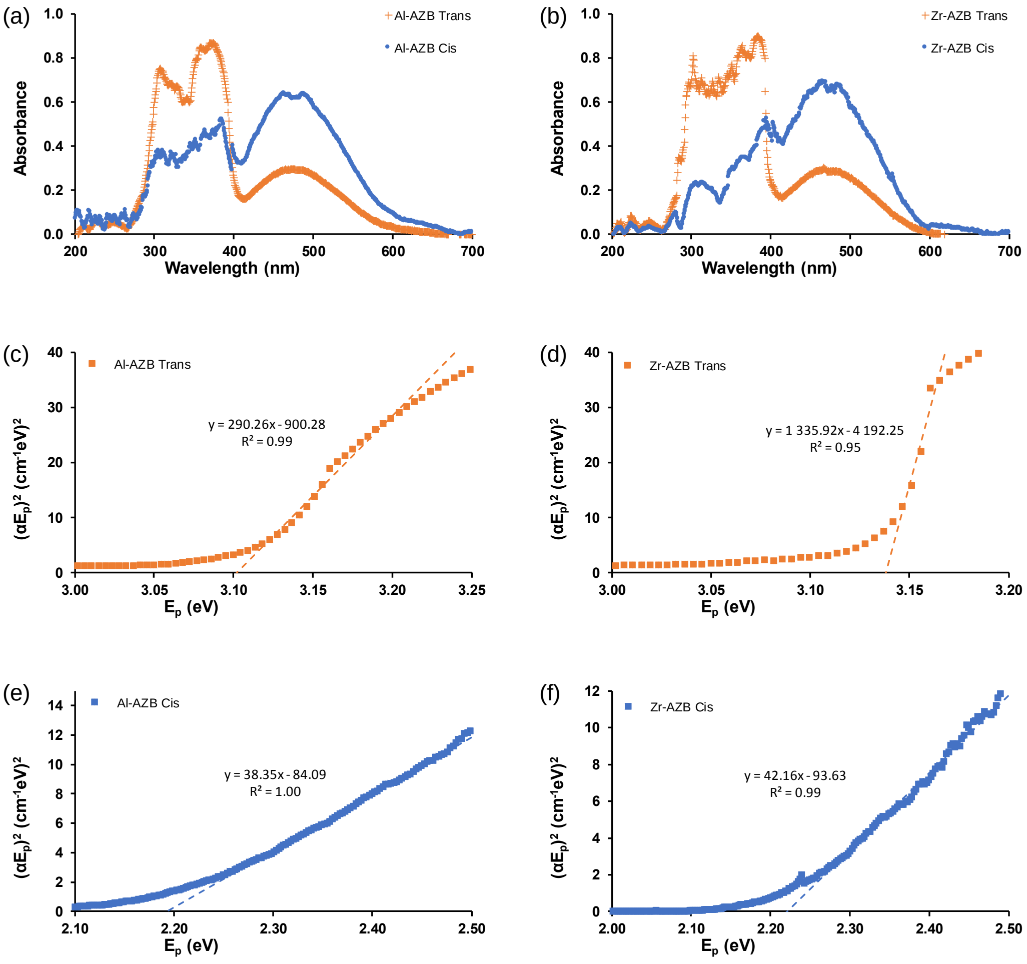

Figure S 9. The UV-Vis spectra in $CHCl_3$ of *trans* and *cis* form of **a)** Al-AZB and **b)** Zr-AZB as well as Tauc plots of **c)** Al-AZB *trans* isomer, **d)** Zr-AZB *trans*, **e)** Al-AZB *cis* and **f)** Zr-AZB *cis*.

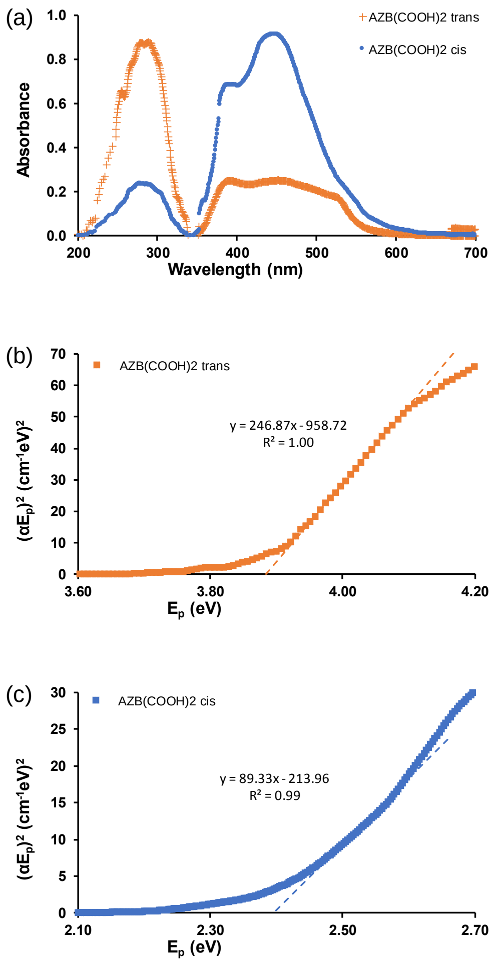

Figure S 10. The UV-Vis spectra in water of *trans* and *cis* form of **a)** AZB(COOH)$_2$ as well as Tauc plots of **b)** AZB(COOH)$_2$ *trans* isomer, **c)** AZB(COOH)$_2$ *cis*.

References

[1] R. Mogale, K.G. Akpomie, J. Conradie, E.H.G. Langner, Dye adsorption of aluminium- and zirconium-based metal organic frameworks with azobenzene dicarboxylate linkers, J. Environ. Manage. 304 (2022) 114166. doi:10.1016/j.jenvman.2021.114166.
